# Hydrogels in the treatment of rheumatoid arthritis: drug delivery systems and artificial matrices for dynamic in vitro models

**DOI:** 10.1007/s10856-021-06547-1

**Published:** 2021-06-22

**Authors:** Isabel Maria Oliveira, Diogo Castro Fernandes, Ibrahim Fatih Cengiz, Rui Luís Reis, Joaquim Miguel Oliveira

**Affiliations:** 1 3B’s Research Group, I3Bs—Research Institute on Biomaterials, Biodegradables and Biomimetics of University of Minho, Headquarters of the European Institute of Excellence on Tissue Engineering and Regenerative Medicine, Avepark, Parque de Ciência e Tecnologia, Zona Industrial da Gandra, 4805-017 Guimarães, Portugal; 2 ICVS/3B’s—PT Government Associate Laboratory, 4805-017 Braga/Guimarães, Portugal

## Abstract

Rheumatoid arthritis (RA) is an autoimmune and chronic inflammatory disorder that mostly affects the synovial joints and can promote both cartilage and bone tissue destruction. Several conservative treatments are available to relieve pain and control the inflammation; however, traditional drugs administration are not fully effective and present severe undesired side effects. Hydrogels are a very attractive platform as a drug delivery system to guarantee these handicaps are reduced, and the therapeutic effect from the drugs is maximized. Furthermore, hydrogels can mimic the physiological microenvironment and have the mechanical behavior needed for use as cartilage in vitro model. The testing of these advanced delivery systems is still bound to animal disease models that have shown low predictability. Alternatively, hydrogel-based human dynamic in vitro systems can be used to model diseases, bypassing some of the animal testing problems. RA dynamic disease models are still in an embryonary stage since advances regarding healthy and inflamed cartilage models are currently giving the first steps regarding complexity increase. Herein, recent studies using hydrogels in the treatment of RA, featuring different hydrogel formulations are discussed. Besides, their use as artificial extracellular matrices in dynamic in vitro articular cartilage is also reviewed.

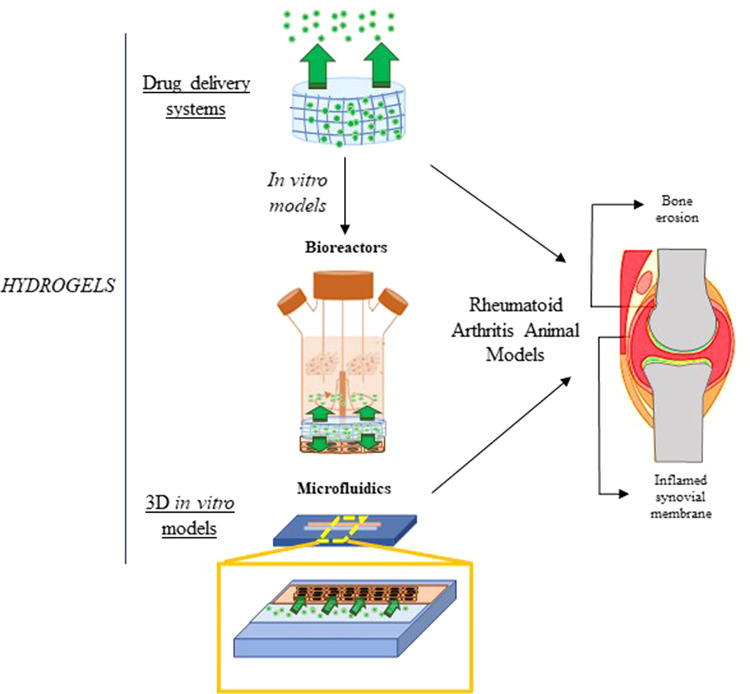

## Introduction

Hydrogels are three-dimensional (3D) networks of hydrophilic polymers able to absorb vast amounts of biological fluids or water [1, 2]. Hydrogels are formed by physical and chemical crosslinking. Physically crosslinked hydrogels are produced through molecular entanglements, ionic, hydrogen bonding, or hydrophobic forces. These hydrogels are structurally weak, and their gelation is reversible. Chemical hydrogels are covalently crosslinked by redox reactions, photo-polymerization, Michael reactions, enzymatic reactions, or disulfide-forming reactions, which are strong and irreversible bonds [3, 4]. Hydrogels, according to the source, can be classified into the natural and the synthetic group [5]. Natural and synthetic polymers present advantages and disadvantages, to increase the physicochemical and biological properties, several materials can be used together [6].

**Fig. 1:**
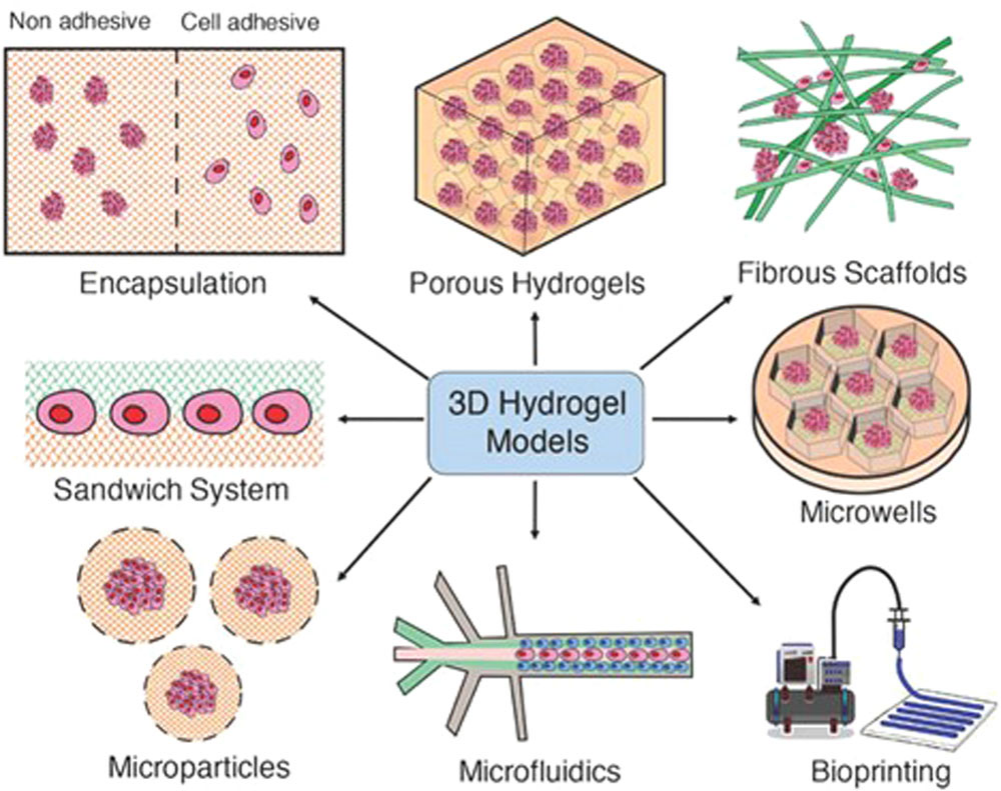
Schematic illustration of the 3D hydrogel models, including hydrogels (encapsulation), porous hydrogels, fibrous hydrogel scaffolds, hydrogel sandwich systems, microwells, hydrogel microparticles, microfluidics, and bioprinted scaffolds. Reprinted from publication [12], Copyright (2019) with permission from John Wiley and Son

Experiments with 2D cell cultures are the usual practice in cell-based assays for various biomedical research purposes, presenting, however several limitations. This cell culture system is incapable of reproducing the anatomical and biochemical properties of tissues and organs [7]. Therefore, the development of model systems that better mimic the physiological microenvironment of the tissue and disease, such as stiffness, topography, and biochemistry, with the capacity to execute prolonged culture tests while maintaining tissue function is required [8]. In vivo, cells are embedded by extracellular matrix (ECM) that plays an important role in several cellular processes such as regulating growth and cell–cell communication and assembling cells into various tissues and organs. So, developing an in vitro cell culture environment that mimics the native ECM is required [9]. Hydrogels are one of the leading biomaterials used and appropriate options due to their unique properties, high water content, porosity, and flexibility, they can mimic the native ECM. Additionally, hydrogels do not affect the metabolic processes of living organisms, and metabolites can pass easily through the hydrogels [10]. So, such an engineered native-like ECM is most likely to offer cells with rational indications for diagnostic and therapeutic investigations. Due to these appealing features, an extensive range of 3D hydrogel platforms have been developed to mimic better the natural tissue environment in vitro [11]. Traditional approaches include cellular encapsulation into hydrogels, cell seeding on porous, and fibrous hydrogels. However, advanced hydrogel platforms have emerged to use hydrogels in functional tissue models, including hydrogel microspheres, hydrogel sandwich systems, hydrogel-based microwells, and 3D bioprinted tissue–hydrogel. Furthermore, hydrogels are usually incorporated within culture platforms such as transwell microfluidic devices (Fig. 1) [12]. Hydrogels have been used to develop a wide range of tissue and disease models. These 3D networks of hydrophilic polymers can undergo physiological swelling and have the mechanical behavior desired for use as an articular in vitro model [13].

**Fig. 2:**
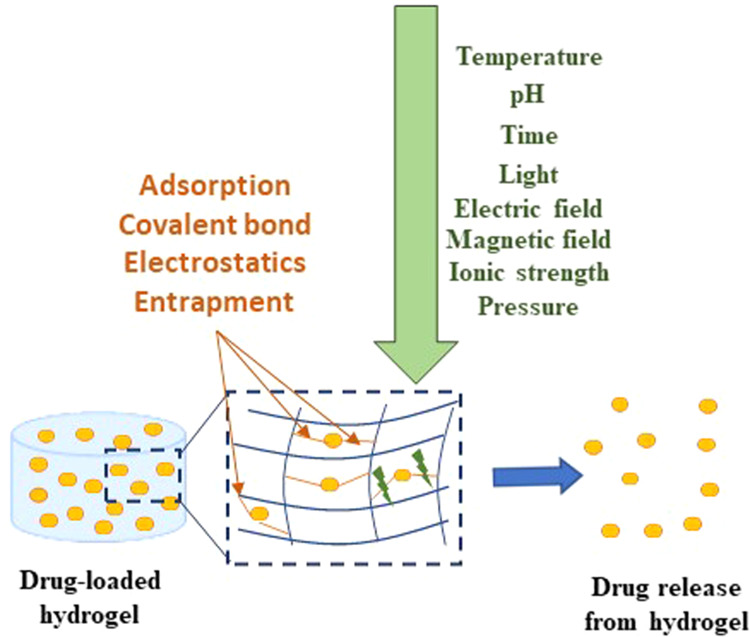
Drug delivery hydrogel in response to several physical and chemical stimuli

The unique physical properties of hydrogels (high porosity, controlled drug release, biocompatibility, biodegradation, and flexibility) make this system an appealing platform for drug delivery applications [2]. The porosity of hydrogels allows the encapsulation of drugs into the gel and consequent drug release at a rate dependent on the diffusion coefficient of the molecule by the hydrogel network [14]. The hydrogels can be produced in a way in which drugs are released slowly, keeping a high local concentration of the drug in the place where they are administrated for a long time [15, 16]. Hydrogels are also biocompatible due to their high content and structural and mechanical similarity of hydrogels to the ECM. The biodegradation pattern is an essential parameter for a drug delivery system. The controlled drug release and degradation profile may be design into hydrogels via the hydrolytic, environment (pH, temperature), or enzymatic pathways (Fig. 2) [17–20].

The administration of hydrogels is also versatile, for example, through oral and topical administration, implantation, or it can be injected [21]. The selection of delivery method is based on the improvement of the overall efficacy and patient compliance.

Drug release through topical route offers several advantages and novel approach. These potential advantages include improved patient compliance, ease of application to the skin, and allow to deliver drugs more selectively to a specific local of action. The main disadvantages arise when the skin acts as a natural barrier that becomes difficult for most drugs to penetrate [22]. Besides that, the scaffolds can be implanted on a site-specific to a more controlled release of active compounds. This allows to preventing side effects caused by high doses and frequent administration. However, implantable hydrogels are a more invasive and painful method with a higher cost [14, 23]. Injectable hydrogels demonstrate promising properties as carriers for targeted drug delivery. They are attracting more attention because they are more comfortable, less painful, have a faster recovery period, lower costs, and present fewer complications and side effects [24].

Rheumatoid Arthritis (RA) is an autoimmune and chronic inflammatory condition. The cause of RA remains poorly known but can involve genetic and environmental factors [25, 26]. The pathology of RA is characterized by infiltration of several inflammatory cells into the synovial membrane and by subsequent cartilage and bone tissue destruction [27, 28]. The pathogenesis begins with the activation of immune cells that promotes the production of pro-inflammatory cytokines which support cell–cell communication in immune response and stimulate the movement of inflammatory cells toward to synovial membrane [29]. The inflammation of the synovium invades adjacent cartilage promoting articular destruction. The articular damage is featured by a hypoxic environment and angiogenesis [29]. The clinical manifestations comprise redness, swelling, and limitation the range of motion. Currently, several treatments are available to relieve pain and control inflammation. However, traditional drug administration often involves high dosages or frequent administration to promote a therapeutic effect, which can decrease overall efficacy and result in severe side effects (heart problems, kidney damage, diabetes, lung infections, among others) [30].

Drug delivery systems are emerging to address existent failures in traditional systems. The advantages of these new approaches are the capacity to deliver a drug more selectively to a specific site allowing more accurate and less frequent dosing. Furthermore, decrease variability in systemic drug concentration, the absorption is more consistent with the site and action mechanism, and reduces the side effects of therapeutics.

Hydrogels are a particularly appealing type of drug delivery system to ensure these disadvantages are minimized and the therapeutic benefits from the drug are optimized [16, 31].

## Hydrogels for the treatment of RA

Several treatments, including non steroidal inflammatory drugs (NSAIDs), glucocorticoidds (GCs), disease-modifying anti-rheumatic drugs (DMARDs) and biological agents, are available to relieve pain and control inflammation with the final goal to achieve disease remission [32]. NSAIDs such as ibuprofen and indomethacin have both analgesic and anti-inflammatory properties but do not change the course of the disease of RA or prevent joint destruction. Most commonly, NSAIDs are available as oral form [33]. GCs such as Betamethasone and Dexamethasone present anti-inflammatory and immunoregulatory activity. They can inhibit neutrophil migration to sites of inflammation and interfere with cytokine stimulation. GCs can be administered orally, intravenously, or intra-articular injection [34]. DMARDs such as Penicillamine and Hydroxychloroquine include a large group of drugs that reduce the progression of joint erosion. Although both NSAIDs and DMARDs agents improve symptoms of active RA, only DMARDs agents have shown to alter the disease course. These therapies can be administrated orally or subcutaneously [35]. Biological agents such as Etanercept and Infliximab are a class of drugs that act by decreasing the inflammatory response in affected joints. Biological agents target specific components of the immune system that play an important role in the sustenance of the disease process and tend to work more quickly than conventional DMARDs. The most of biological agents are currently administrated through subcutaneous/intramuscular injection [36].

Current strategies for the treatment of RA can decrease inflammation in the joints, alleviate pain, and slow down joint damage; however, the treatments are associated with poor pharmacokinetic distribution to the specific site of disease, short half-life, and several side effects [37]. The hydrogels offer suitable drug delivery vehicles to guarantee that the handicaps of traditional drugs are reduced, and the therapeutic effects from the medications are maximized.

Several studies report the potential application of hydrogels in the treatment of RA. In this section, the studies provided were selected among the more promising strategies using hydrogels in the treatment of RA summarized (Table 1).

**Table 1:** Hydrogels for the delivery of drugs/bioactive agents used in the treatment of RA

Delivery vehicle	Drugs/Agents	Conditions	Main results	Reference
Chitosan hydrogels combined with alginate microspheres	Diclofenac	In vivo study New Zealand rabbits (3 weeks post-treatment)	The anti-inflammation efficacy of the combined hydrogels in rabbits with RA was higher than that of drug solution and pure chitosan hydrogels	[38]
Cationic agarose hydrogels	ASO	In vivo study AA CLA CIA	Alleviating inflammation and tissue destruction were demonstrated in more than 90% of the testing animals reduction of central inflammatory cytokines and decrease of joint swelling and tissue damage	[43]
Fibrin gel (FGB) and PLGA-PEG-PLGA hydrogel (HGB)	BMMSCs	In vivo study OVA rabbits (12 weeks post-treatment)	The administration of BMMSCs reduced inflammatory cytokine levels and improved joint swelling in both groups The preservation of adjacent cartilage and enhanced cartilage repair were detected	[47]
Nanofibrous hydrogel	MMP-2 and MMP-9	In vivo study Balb/C mice (8 weeks post-treatment)	The results showed that nanofibrous gels could persist stably after injection into healthy joints of mice in vitro studies showed that release loaded agents in response to synovial fluid from arthritic patients	[44]
Microemulsion-based topical hydrogels	TNX	Ex vivo study Laca Mice Sprague–Dawley (48 h post-treatment)	Microemulsion formulations demonstrated be superior in controlling inflammation as compared to traditional topical dosage forms and presented efficacy equivalent to the oral formulation	[48]
NLCs	MTX	In vivo study Wistar rat (24 h post-treatment)	The immunocytochemistry to detect IL-6 expression and immunofluorescence assay showed that promoted apoptosis occurred in an in vitro arthritis model treated with NLCs-MTX. It was verified decreased inflammation and activated apoptosis promoted by MTX encapsulated NLCs in rheumatoid arthritic cells Histopathological analysis of skin suggested the safety potential of NLCs	[49]
MTX-NLCs + CE hydrogel	MTX	In vivo study CFA induced arthritis rat (17 weeks post-treatment)	MTX-NLCs + CE hydrogel significantly decreases the inflammation in RA animal model	[50]
Lipogel and hydrogel microemulsion	Diflunisal	Ex vivo study Human abdominal skin (24 h post-treatment)	The lipogels delivered a significant amount of drug through the skin than the hydrogel. The constitution of lecithin showed to affect the skin permeability increasing the capability of the lipogel	[51]
TG-18 hydrogel	TA	In vivo study K/BxN serum-transfer model of IA (14 days post-treatment)	In arthritic mice, hydrogel encapsulated with a fluorescent dye showed flare-dependent disassembly assessed as a loss of fluorescence. A unique dose of TA-encapsulated hydrogel decreased arthritis manifestation in the injected paw	[46]
Aspasome hydrogel	MTX	In vivo study AIA in Wistar rats (21 days post- treatment)	Transdermal application of methotrexate-loaded aspasome hydrogel in model disease demonstrated a more significant decrease of rat paw diameter, tumor necrosis factor-alpha (TNF-α), interleukin (IL-1) β, cartilage damage, inflammation, pannus formation, and bone resorption when compared to arthritic control rats The group treated with free methotrexate exhibited intermediate effects however, the group treated with free aspasome did not show to have an effect	[52]
Propyl-capped PCLA-PEG-PCLA gel	Celecoxib (40 mg/g and 120 mg/g)	In vivo study Dutch Warmblood horses (5 weeks post-treatment)	One intra-articular injection of LCLB-gel or HCLB-gel demonstrated a sustained and controlled intra-articular release in healthy and inflamed joints The celecoxib formulations presented a soft effect on inflammatory and synovial fluid biomarkers, but these returned to threshold 1 week after administration high levels of celecoxib were detected in the joint after 1 month, but no overall anti-inflammatory effects was observed, maybe due to the moderate synovitis There were no adverse effects during the study period	[39]
Scv gel	NO	In vivo study collagen-induced arthritis mouse model (35 days post- treatment)	The NO-Scv gel decreased inflammation levels and showed good biocompatibility Therapeutic effect of the NO-Scv gel in decreasing the onset of RA is observed in a mouse RA model when compared to the effects of dexamethasone alone	[40]
DLTH with chitosan–glycerin–borax	Dexamethasone	In vivo study Collagen-induced arthritis wistar rats (3 weeks post-treatment)	Paw swelling, arthritis scores, and joint inflammation destruction were reduced in the group treated with DLTH DLTH demonstrated down-regulated serum IL-17A and mRNA levels of inflammatory factors DLTH-treated rats elucidated the pain-reducing effects of DLTH	[41]
Hydrogel formulations (Poloxamer 407 and chitosan) loaded PLGA and PCL nanoparticles	DS	In vitro study	After 1-month prolonged in vitro release of DS was reached by using polymeric nanoparticles with in situ hydrogels	[42]
HA-AC hydrogels	Iguratimod	In vivo study CIA rats (21 days post-treatment)	The results demonstrated superior bioavailability and longer half-life time with NanoIGUR-loaded hydrogel than traditional iguratimod Animal experiments for 21 days showed that subcutaneous injection of NanoIGUR-loaded hydrogel (10 mg/kg every 3 days) and traditional iguratimod (10 mg/kg daily) exhibited identical efficacy in diminishing arthritis index score, pathological score, and expression of inflammatory cytokines	[45]

**Fig. 3:**
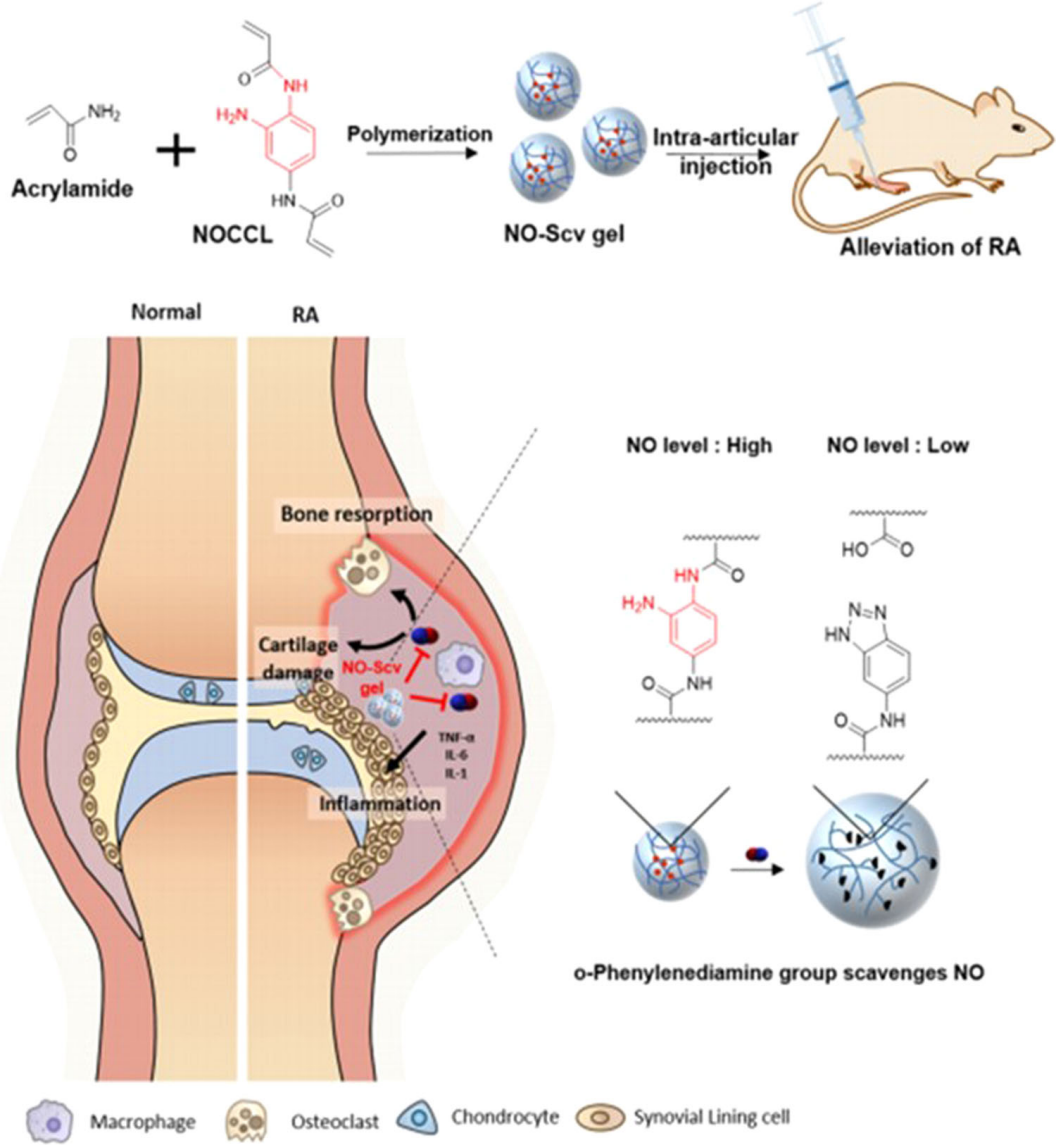
Schematic Illustration of intra-articular injection of NO-Scv Gel in suppressing of RA in a mouse model. Reprinted with permission from [40]. Copyright (2019) American Chemical Society

The most common techniques are based on the use of injectable hydrogels to treatment of RA. Qi et al. [38] developed intra-articular administrated chitosan thermosensitive hydrogels with diclofenac sodium (DS)-loaded alginate microspheres to assess the potential of hydrogels as drug delivery systems for promoting the anti-inflammatory effect. The results showed that the anti-inflammatory efficacy of hydrogels after 3 weeks was higher than that of diclofenac solution and chitosan hydrogels alone. A study performed by Cokelaere et al. [39] evaluated the administration intra-articular sustained of two formulations of celecoxib (40 and 120 mg/g) in a poly(ε-caprolactone-*co*-lactide) PCLA-PEG-PCLA triblock copolymer in an equine repeated lipopolysaccharide (LPS) synovitis model. Only one intra-articular injection of the low dose (LCLB)-gel or high dose (HCLB)-gel demonstrated a sustained and controlled intra-articular release in healthy and inflamed joints. The celecoxib formulations presented a soft effect on inflammatory and synovial fluid biomarkers, but these returned to the threshold 1 week after administration. High levels of celecoxib were detected in the joint after 1 month, but no overall anti-inflammatory effects were observed, maybe due to the moderate synovitis. Furthermore, there were no side effects during the study period. Therefore, this approach should be assessed for its impact on longer-term relief of inflammatory joint pain. Yeo et al. [40] evaluated the capability of intra-articular injection of the nitric oxide (NO)-scavenging nanogel (NO-Scv gel) to treat RA. After 35 days, the NO-Scv gel decreased inflammation levels and showed good biocompatibility. Moreover, the therapeutic effect of the NO-Scv gel in diminishing the onset of RA is observed in a mouse RA model when compared to the effects of dexamethasone alone (Fig. 3). Wang et al. [41] produced a sustained release formulation-intra-articular injectable dexamethasone-encapsulated thermosensitive hydrogel (DLTH) chitosan–glycerin–borax as the carrier for the suppression of inflammation and pain in collagen-induced arthritis (CIA) rats. The data showed that paw swelling, arthritis scores, and joint inflammation destruction were reduced in the group treated with DLTH after 3 weeks. DLTH demonstrated down-regulated serum IL-17A and mRNA levels of inflammatory factors. Furthermore, DLTH-treated rats elucidated the pain-reducing effects of DLTH. So, these results suggested that DLTH joint injection prevents synovial inflammation. A study made by Kuçukturkmen et al. [42] analyzed the effect of in situ gelling hydrogel formulations (Poloxamer 407 and chitosan) containing DS-loaded Poly(lactic-co-glycolic acid) (PLGA) and poly(ε-caprolactone) (PCL) nanoparticles for intra-articular injection. After 1-month prolonged in vitro release of DS was reached by using polymeric nanoparticles with in situ hydrogels.

A study made by Dong et al. [43] produced cationic agarose injectable hydrogels to deliver antisense oligodeoxynucleotides (ASOs) targeting the mRNA of TNF-α. Different kinds of animal models were used to assesses the therapeutic benefits of ASO-Gel, including CIA, carrageen/LPS-induced arthritis, adjuvant-induced arthritis (AA), and models. After 28 days of treatment, the effects of ASO-c-agarose in decreasing inflammation and tissue destruction were demonstrated in most tested animals, with reduction of main inflammatory cytokines and decrease of joint swelling and tissue destruction. Another study reported the production of an injectable self-assembled nanofibrous hydrogel, which can encapsulate and release agents in response to matrix metalloproteinases (MMP-2 and MMP-9) that are upregulated in RA. The results showed that nanofibrous gels could persist stably after 8 weeks of injection into healthy joints of mice, and in vitro studies showed that release loaded agents in response to synovial fluid from arthritic patients [44]. Another study investigated the effect of nanoiguratimod-loaded hyaluronic acid–acrylate hydrogel (NanoIGUR-loaded hydrogel) composites in CIA rats to improve the bioavailability of drug and to alleviate side effects through the sustained release of therapeutics [45]. The results demonstrated superior bioavailability and longer half-life time with NanoIGUR-loaded hydrogel than traditional iguratimod. Animal experiments for 21 days showed that subcutaneous injection of NanoIGUR-loaded hydrogel (10 mg/kg every 3 days) and traditional iguratimod (10 mg/kg daily) exhibited identical efficacy in diminishing arthritis index score, pathological score, and expression of inflammatory cytokines [45]. Joshi et al. [46] reported the production of a triglycerol monostearate (TG-18) injectable hydrogel encapsulated with triamcinolone acetonide (TA) releases after being in contact with enzymes or synovial fluid from patients with RA. In arthritic mice, hydrogel encapsulated with a fluorescent dye showed flare-dependent disassembly assessed as a loss of fluorescence. Furthermore, a unique dose of TA-encapsulated hydrogel decreased arthritis manifestation in the injected paw after 14 days.

Besides injectable hydrogels, the implanted and topical hydrogels are widely used in the treatment of RA. Liu et al. [47] developed a fibrin gel- and poly(l-lactide-co-glycolide)-poly(ethylene glycol)-poly(l-lactide-co-glycolide) hydrogel-assisted bone marrow mesenchymal stem cells (BMMSCs) referred to FGB and HGB groups, respectively, to be intra-articullary transplanted into subchondral defects of ovalbumin-induced arthritis in rabbits for the treatment of antigen-induced arthritis. The BMMSCs have an important role in secreting soluble factors that promote tissue regeneration. Furthermore, these cells provide an immunoregulatory ability and induce immunosuppressive effects demonstrated in several autoimmune diseases. The administration of BMMSCs reduced inflammatory cytokine levels and improved joint swelling in both groups, after 12 weeks. Furthermore, the preservation of adjacent cartilage and enhanced cartilage repair was detected. The results showed that HGB group presented a better therapeutic benefit than the FGB group. Goindi et al. [48] developed microemulsion-based topical hydrogels of Tenoxicam (TNX) to treat arthritis. In vivo anti-inflammatory and anti-arthritic activity of the TNX formulations was assessed using several inflammatory models. Microemulsion formulations demonstrated to be better in controlling inflammation than traditional topical forms and presented efficacy similar to an oral formulation. A study made by Garg et al. [49] investigated the use of nanostructured lipid carriers (NLCs) produced through lipid mixture and chemical permeation increaser-based hydrogel for an effective transdermal delivery of methotrexate (MTX) to induce apoptosis of RA. The immunocytochemistry to detect IL-6 expression and immunofluorescence assay showed that promoted apoptosis occurred in an in vitro arthritis model treated with NLCs-MTX. It was verified decreased inflammation and activated apoptosis promoted by MTX encapsulated NLCs in rheumatoid arthritic cells. Furthermore, histopathological analysis of rat skin suggested the safety potential of NLCs after 24 h. Posteriorly the same group [50] evaluated the effect of MTX encapsulated NLCs and chemical enhancer co-incorporated hydrogel (gel-(MTX-NLCs + CE)) for competent transdermal delivery of MTX in a complete Freund’s adjuvants (CFA)-induced arthritis in rats. The gel-(MTX-NLCs + CE), gel-MTX, gel-(MTX-NLCs) was applied on paws and ankles of CFA induced arthritis rat model once a day for 17 weeks. Results showed that the transcutaneous ability of MTX-loaded NLCs and CE co-incorporated hydrogel significantly reduced the inflammation in the RA animal model. Sallam et al. [51] developed a lecithin organogels transdermal delivery system for diflunisal and studied human skin penetration capability compared to optimized microemulsion-based hydrogel. The lipogels delivered a significant amount of drug through the skin than the hydrogel after 24 h. The constitution of lecithin showed to affect the skin permeability increasing the capability of the lipogel. Another study developed MTX aspasomes encapsulated into a hydrogel and tested in AIA model in Wistar rats [52]. Transdermal application of MTX-loaded aspasome hydrogel in model disease demonstrated a more significant decrease of rat paw diameter, tumor necrosis factor-alpha (TNF-α), interleukin (IL-1) β, cartilage damage, inflammation, pannus formation, and bone resorption when compared to arthritic control rats after 21 days. Furthermore, the group treated with free MTX exhibited intermediate effects however, the group treated with free aspasome did not show to have an effect. The results demonstrated that drug-loaded therapeutically active carrier system presented a non-invasive controlled release transdermal formulation with good drug encapsulation, drug permeation rate, and showed a more effective therapeutic effect in treatment of RA than the drug alone [52].

The list of U.S. Food and Drugs Administration approved drugs for RA is still limited [37, 53], and RA is still an incurable disease with a high impact on the patient’s lifestyle. The drug development process has systemic problems, several of them related to the use of animal (non-human) models. This problem, alongside its alternatives, is discussed in the following section, reviewing the use of hydrogels as artificial matrices to develop cartilage disease models.

## Hydrogels as artificial extracellular matrices for dynamic in vitro models

The process of therapeutic approval is long from in vitro tests to widespread medical practice. With the increased costs of drug development, the paradigm of using animal experimentation as a predictor of results in humans has been questioned [54, 55]. Animal models are considered the gold standard in preclinical studies of pathophysiological mechanisms of RA. They present many similarities with human arthritic diseases and are widely used for testing new therapeutic strategies [56, 57]. However, the animal models present some limitations, such as limited development of arthritis, pathophysiology in animals does not completely mimic the human pathogenic disease and, are inadequate for high drug screening [58].

After revising several systematic reviews evaluating results from animal experimentation, during the last two decades several publications have emphasized the shortcomings of animal testing [54, 59–62]. A group of academia and industry experts presented recently an alternative for the near future to animal models: human-based microphysiological models, developed with the principles of tissue engineering. These systems have the advantage to have human cells, annulling the issue of between species results translation. However, it carries several engineering challenges, mostly bound to organs’ complexity and systemic dynamics [63].

To produce a fully biomimetic model of a joint’s cartilage it is necessary to ensure that these dynamics are transposed from in vivo to in vitro [64].

A biomimetic dynamic articular cartilage model must include a biocompatible hydrogel, with physiological values of physical and biochemical cues for chondrogenesis; multi-axial mechanical stimulation, mimicking the articulated limbs; and physiological synovial fluid shear stress, transporting nutrients and regulating the soluble oxygen levels [65]. To have an improvement on the outcome of drug development, existing such models, with throughput and scale-up capacity in a near future, it is necessary that innovative therapeutics systems are developed meanwhile [66].

The near-physiological dynamic systems most commonly used are Gel-based systems as artificial extracellular matrices combined with a dynamic environment, bioreactors, or microfluidic devices [67, 68].

Several studies using hydrogels as ECM and bioreactors for articular cartilage was explored deeper its tissue-specific particularities. All of the mentioned models are 3D cultured using biomaterials to support the growth and physiologic biomechanics of tissue cells. For this purpose, these biomaterials are processed into biocompatible hydrogels or scaffolds. Biocompatibility implies biofunctionality, meaning that these biomaterials not only must promote cell viability and growth but also promote tissue-specific phenotype and physiology. Thus, the selection of the biomaterials used is crucial for the development of a representative model.

**Fig. 4:**
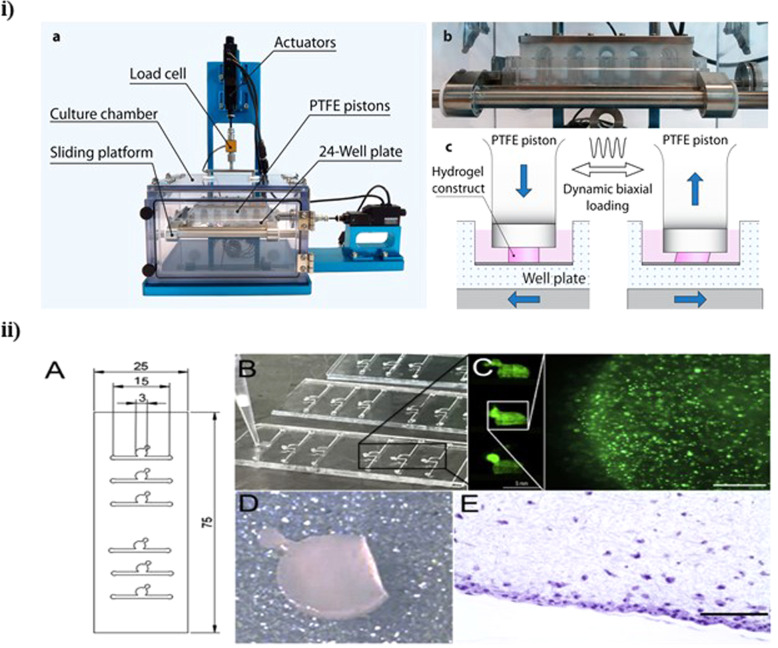
(i) Mechanical stimulation bioreactor system. (a) Front view depicting the bioreactor stand (anodized in blue) and the removable polycarbonate culture chamber. (b) Close-up view of PTFE pistons lowered into a standard 24-well plate on the actuator-driven sliding platform. (**c**) Shear deformation of hydrogel construct fully immersed in culture media. Reprinted with permission from [69]. (ii) (**A**) CAD design of cartilage-on-a-chip device. (**B**) Photograph of an actual cartilage-on-a-chip device showing loading of cell-laden hydrogel in the top chamber. (**C**) Overview picture of three cell culture chambers and right side showing one individual culture chamber featuring CMFDA-stained primary equine chondrocytes cultivated on-chip. (**D**) Intact cell-laden fibrin hydrogel clot released from the device before downstream analyses and (**E**) histological section of chondrocytes-on-a-chip. Reprinted from publication [75], Copyright (2021) with permission from Elsevier

To improve clinical outcomes and simplify a larger use of engineered tissues, bioreactor systems able of increasing and monitoring neotissues is needed. A study made by Meinert et al. [69] developed an innovative bioreactor system (Fig. 4i) able to applying specific uni- or biaxial mechanical stimulation to developing cartilage neotissues in a fully controlled and automated way to improve the quality of biological implants and decrease the manufacturing costs. They investigated the effects of pre-culture and various uni- and biaxial loading regimes on human chondrocyte gene expression in gelatin methacryloyl and hyaluronic acid methacrylate hydrogels. The results showed that uniaxial shear and compression, as well as biaxial stimulation, induce the expression of chondrogenic marker genes. Furthermore, showed that mechanostimulation of tissue-engineered constructs consisting of clinically relevant cells and biomaterials increases the biosynthesis and accumulation of hyaline cartilage-specific ECM, producing neotissues with higher native-like biochemical properties [69]. Another study [70] investigated the effect of introducing gradients of interstitial flow on chondrocyte-seeded agarose hydrogels using a bioreactor. They observed that flow stimulation of chondrocyte-seeded agarose hydrogels allows the enhance of glycosaminoglycans and type II collagen deposition in the surface region of the hydrogel exposed to flow. Furthermore, it was observed that interstitial flow increases convective mass transport independently of molecular size inside the boundary layer closer to hydrogel surface and that the convective contribution to transport decreases with depth in connection with interstitial flow gradients [70]. A study made by Daly et al. [71] investigated if dynamic bioreactor culture, at specific oxygen conditions, could expedite the development of large cartilage tissues using mesenchymal stem cell loaded alginate hydrogels. The dynamic culture conditions were investigated by performing the test at 20% O_2_ and 3% O_2_. At 20% O_2_, dynamic culture substantially restrained chondrogenesis in engineered tissues of all sizes. However, at 3% O_2_ dynamic culture considerably increased the distribution and amount of cartilage matrix components, collagen II and sulfated glycosaminoglycan and collagen II when compared to static conditions. So, these results showed that dynamic culture systems that offer suitable nutrient disposal and a low oxygen environment can be used to engineer large homogeneous cartilage tissues [71].

Bioreactors, despite allowing real-size models, using multi-stimuli setups, have a lesser throughput than miniaturized models, namely microphysiological systems that decrease the volume of consumables, decrease sample size, and increase monitoring capacity. Organ-on-a-chip technology has been more common in recent years and has been applied to in vitro development of cartilage models in the most recent years [72, 73]. These models show that this technology already allows in vitro model production and maintenance [72, 74], and explant culture [73].

Several studies show that microfluid-based chondrocyte systems may present a new platform for future investigation in the behavior of differentiated chondrocytes. A study made by Rosser et al. [75] developed a 3D chondrocyte on-a-chip model mimicking in vivo articular chondrocyte morphology, cell distribution, metabolism, and gene expression (Fig. 4ii). This model was established by loading hydrogel-embedded primary (equine) chondrocytes and a physiologic nutrient diffusion gradient across the simulated matrix. The results showed that chondrocytes remained viable with high Sox9, aggrecan, and Col2 expression normal of articular chondrocytes. Furthermore, the chondrocytes were exposed to pro-inflammatory cytokines to mimic the in vitro osteoarthritis model and the results demonstrated that on-chip established equine cartilage react to biochemical injury and respond to steroid treatment [75]. Another study [76] designed a microfluidic agarose-based construct and established bioprocessing conditions to meet nutrient transport requirements of a large, full-thickness articular cartilage construct. The results showed that microfluidic agarose-based hydrogels approaching thicker and more robust constructs allowed improve proliferation and matrix deposition but not apparent mechanical properties suggesting that this platform has clinical utility [57]. A study made by Li et al. [76] developed a facile, very low cost-based microfluidic platform to produce visible light-cured microgels composed of gelatin norbornene (GelNB) and a poly(ethylene glycol) (PEG) cross-linker. The microfluidic device was designed to encapsulate human bone marrow-derived mesenchymal stem cells (hBMSCs) and for be used articular cartilage tissue regeneration. The results demonstrated that the process allows the rapid in situ microencapsulation of hBMSCs under biocompatible microfluidic-processing conditions. The hBMSCs presented a remarkably high degree of chondrogenesis in the GelNB microgels with chondro-inductive media, particularly regarding the hyaline cartilage structure, with substantial upregulation in type II collagen expression compared to the bulk hydrogel and standard culture [76].

Besides the use of hydrogels as ECM in dynamic in vitro models, the use of hydrogels as bioinks for bioprinting of in vitro models has shown a great potential for cartilage tissue engineering. The dynamic microenvironment of ECM is not totally mimic from the traditional static environment of the hydrogels and, 3D bioprinting allows adaptability, architecture control, and repeatability that can overcome the limits of traditional biofabrication systems [77]. The selection of biomaterials for the printing mostly depends on their biocompatibility with cell growth and function and also their printing characteristics, extrudability, post-printing stability, such as viscosity [78, 79]. After revising several systematic reviews the most of the published works use a limited range of bioinks, including gelatin, collagen, alginate, modified copolymer PEG, hyaluronic acid, and photocurable acrylates/methacrylates to cartilage tissue engineering [78, 80]. The development of these innovative bioinks gives to biomedical engineering community closer to expectations of fabricated structures of ideal properties able of replicating native tissues, while further improve regeneration and therapeutic handicaps [77].

## Conclusion and future perspectives

RA is a highly debilitating chronic autoimmune disease that typically causes pain, swelling, and stiffness in the joints. Although there are several treatments that allow an improvement in the physical condition of patients, most of them are not completely effective and have several serious side effects. Several therapeutic approaches have been developed over the past few years to improve efficacy and decrease side effects caused by drugs. Hydrogels have a unique combination of characteristics, high porosity, biocompatibility, biodegradation, and flexibility make them useful in drug delivery applications. Through the literature review where hydrogels were used to treat RA, it was possible to verify that this drug delivery platform was able to potentiate the effect of drugs when compared with traditional drugs, both in vitro and in vivo studies. Despite the advances reached in the treatment of RA, it is important to fill some gaps namely mimic the dynamic system existing in the human body.

The drug development industry is facing a severe halt, forcing a paradigm change. One of the most blatant reasons for this hampering has been the use of animal experimentation for drug testing. Experts from academia to the industry recognize today that dynamic in vitro human models are the alternative with better prospects since it bypasses several of the problems of animal models.

The studies mentioned above allow demonstrated that Gel-based systems as artificial extracellular matrices combined with dynamic systems based in bioreactors and microfluidic devices are promising preclinical models to articular cartilage, with additional potential to be used in several applications in regenerative medicine.

Considering the current models, diseased articular cartilage models will evolve in the following years for a combination of intensive characterization of the biomaterials, cells, and fluid mechanics employed and biomimetic cellular diversity.

## References

[1] Caló E, Khutoryanskiy VV (2015). Biomedical applications of hydrogels: a review of patents and commercial products. Eur Polym J.

[2] Chai Q, Jiao Y, Yu X (2017). Hydrogels for biomedical applications: their characteristics and the mechanisms behind them. Gels..

[3] Akhtar MF, Hanif M, Ranjha NM (2016). Methods of synthesis of hydrogels: a review. Saudi Pharm J.

[4] Ghasemiyeh P, Mohammadi-Samani S (2019). Hydrogels as drug delivery systems; pros and cons. Trends Pharm Sci.

[5] Ahmed EM (2015). Hydrogel: preparation, characterization, and applications: a review. J Adv Res.

[6] Guo B, Ma PX (2014). Synthetic biodegradable functional polymers for tissue engineering: a brief review. Sci China Chem..

[7] Edmondson R, Broglie JJ, Adcock AF, Yang L (2014). Three-dimensional cell culture systems and their applications in drug discovery and cell-based biosensors. Assay Drug Dev Technol.

[8] Chen F-M, Liu X (2016). Advancing biomaterials of human origin for tissue engineering. Prog Polym Sci.

[9] Urbanczyk M, Layland SL, Schenke-Layland K (2020). The role of extracellular matrix in biomechanics and its impact on bioengineering of cells and 3D tissues. Matrix Biol.

[10] Geckil H, Xu F, Zhang X, Moon S, Demirci U (2010). Engineering hydrogels as extracellular matrix mimics. Nanomedicine.

[11] Lev R, Seliktar D (2018). Hydrogel biomaterials and their therapeutic potential for muscle injuries and muscular dystrophies. J R Soc Interface.

[12] Liaw CY, Ji S, Guvendiren M (2018). Engineering 3D hydrogels for personalized in vitro human tissue models. Adv Healthc Mater.

[13] Chuang E-Y (2018). Hydrogels for the application of articular cartilage tissue engineering: a review of hydrogels. Adv Mater Sci Eng.

[14] Li J, Mooney DJ (2016). Designing hydrogels for controlled drug delivery. Nat Rev Mater.

[15] Hoare TR, Kohane DS (2008). Hydrogels in drug delivery: progress and challenges. Polymer.

[16] Narayanaswamy R, Torchilin VP (2019). Hydrogels and their applications in targeted drug delivery. Molecules.

[17] Slaughter BV, Khurshid SS, Fisher OZ, Khademhosseini A, Peppas NA (2009). Hydrogels in regenerative medicine. Adv Mater.

[18] Mantha S, Pillai S, Khayambashi P, Upadhyay A, Zhang Y, Tao O (2019). Smart hydrogels in tissue engineering and regenerative medicine. Materials (Basel).

[19] Bacelar AH, Cengiz IF, Silva-Correia J, Sousa RA, Oliveira JM, and Reis RL. "Smart" Hydrogels in Tissue Engineering and RegenerativeMedicine Applications", Handbook of Intelligent Scaffolds for Regenerative Medicine, 2nd Edition, Khang G. ed., Pan Stanford Publishing, pp. 334–367, 2017.

[20] Li X, Su X (2018). Multifunctional smart hydrogels: potential in tissue engineering and cancer therapy. J Mater Chem B..

[21] Sosnik A, Seremeta KP (2017). Polymeric hydrogels as technology platform for drug delivery applications. Gels.

[22] Silna E, Krishnakumar K, Nair SK, Narayanan A. Hydrogels in topical drug delivery@ a review. 2016.

[23] Calori IR, Braga G, de Jesus PdCC, Bi H, Tedesco AC (2020). Polymer scaffolds as drug delivery systems. Eur Polym J.

[24] Lee JH (2018). Injectable hydrogels delivering therapeutic agents for disease treatment and tissue engineering. Biomater Res.

[25] Anaya JM, Shoenfeld Y, Rojas-Villarraga A, Levy RA, Cervera R, editors. Autoimmunity: From Bench to Bedside. Bogota (Colombia): El Rosario University Press; 2013. 29087650

[26] Guo Q, Wang Y, Xu D, Nossent J, Pavlos NJ, Xu J (2018). Rheumatoid arthritis: pathological mechanisms and modern pharmacologic therapies. Bone Res.

[27] Clancy J, Hasthorpe H. Pathophysiology of rheumatoid arthritis: nature or nurture? Primary Health Care. 2011;21(9).

[28] Murphy CA, Garg AK, Silva-Correia J, Reis RL, Oliveira JM, Collins MN (2019). The meniscus in normal and osteoarthritic tissues: facing the structure property challenges and current treatment trends. Annu Rev Biomed Eng.

[29] Choy E (2012). Understanding the dynamics: pathways involved in the pathogenesis of rheumatoid arthritis. Rheumatology.

[30] Wen H, Jung H, Li X (2015). Drug delivery approaches in addressing clinical pharmacology-related issues: opportunities and challenges. AAPS J.

[31] Sharpe LA, Daily AM, Horava SD, Peppas NA (2014). Therapeutic applications of hydrogels in oral drug delivery. Expert Opin Drug Deliv.

[32] Bullock J, Rizvi SAA, Saleh AM, Ahmed SS, Do DP, Ansari RA (2018). Rheumatoid arthritis: a brief overview of the treatment. Med Princ Pr.

[33] Crofford LJ (2013). Use of NSAIDs in treating patients with arthritis. Arthritis Res Ther.

[34] Ronchetti S, Ricci E, Migliorati G, Gentili M, Riccardi C (2018). How glucocorticoids affect the neutrophil life. Int J Mol Sci.

[35] Drosos A (2003). Methotrexate intolerance in elderly patients with rheumatoid arthritis: what are the alternatives?. Drugs Aging.

[36] Curtis JR, Singh JA (2011). Use of biologics in rheumatoid arthritis: current and emerging paradigms of care. Clin Therapeutics.

[37] Quan L-D, Thiele GM, Tian J, Wang D (2008). The development of novel therapies for rheumatoid arthritis. Expert Opin Ther Pat.

[38] Qi X, Qin X, Yang R, Qin J, Li W, Luan K (2016). Intra-articular administration of chitosan thermosensitive in situ hydrogels combined with diclofenac sodium-loaded alginate microspheres. J Pharm Sci.

[39] Cokelaere SM, Plomp SGM, de Boef E, de Leeuw M, Bool S, van de Lest CHA (2018). Sustained intra-articular release of celecoxib in an equine repeated LPS synovitis model. Eur J Pharm Biopharm.

[40] Yeo J, Lee YM, Lee J, Park D, Kim K, Kim J (2019). Nitric oxide-scavenging nanogel for treating rheumatoid arthritis. Nano Lett.

[41] Wang QS, Xu BX, Fan KJ, Li YW, Wu J, Wang TY (2020). Dexamethasone-loaded thermosensitive hydrogel suppresses inflammation and pain in collagen-induced arthritis rats. Drug Des Dev Ther.

[42] Küçüktürkmen B, Umut Can Ö, Bozkir A (2017). In situ hydrogel formulation for intra-articular application of diclofenac sodium-loaded polymeric nanoparticles. Turkish J Pharm Sci.

[43] Dong L, Xia S, Chen H, Chen J, Zhang J (2009). Spleen-specific suppression of TNF-alpha by cationic hydrogel-delivered antisense nucleotides for the prevention of arthritis in animal models. Biomaterials.

[44] Vemula PK, Boilard E, Syed A, Campbell NR, Muluneh M, Weitz DA (2011). On-demand drug delivery from self-assembled nanofibrous gels: a new approach for treatment of proteolytic disease. J Biomed Mater Res A.

[45] Ma Z, Tao C, Sun L, Qi S, Le Y, Wang J (2019). In situ forming injectable hydrogel for encapsulation of nanoiguratimod and sustained release of therapeutics. Int J Nanomed.

[46] Joshi N, Yan J, Levy S, Bhagchandani S, Slaughter KV, Sherman NE (2018). Towards an arthritis flare-responsive drug delivery system. Nat Commun.

[47] Liu H, Ding J, Li C, Wang C, Wang Y, Wang J, et al. Hydrogel is superior to fibrin gel as matrix of stem cells in alleviating antigen-induced arthritis. Polymers (Basel). 2016;8. 10.3390/polym8050182. 10.3390/polym8050182 PMC6431989 30979276

[48] Goindi S, Narula M, Kalra A (2016). Microemulsion-based topical hydrogels of tenoxicam for treatment of arthritis. AAPS PharmSciTech.

[49] Garg NK, Tyagi RK, Singh B, Sharma G, Nirbhavane P, Kushwah V (2016). Nanostructured lipid carrier mediates effective delivery of methotrexate to induce apoptosis of rheumatoid arthritis via NF-κB and FOXO1. Int J Pharm.

[50] Garg NK, Singh B, Tyagi RK, Sharma G, Katare OP (2016). Effective transdermal delivery of methotrexate through nanostructured lipid carriers in an experimentally induced arthritis model. Colloids Surf B Biointerfaces.

[51] Sallam MA, Motawaa AM, Mortada SM (2015). An insight on human skin penetration of diflunisal: lipogel versus hydrogel microemulsion. Drug Dev Ind Pharm.

[52] Ghosh S, Mukherjee B, Chaudhuri S, Roy T, Mukherjee A, Sengupta S (2018). Methotrexate aspasomes against rheumatoid arthritis: optimized hydrogel loaded liposomal formulation with in vivo evaluation in wistar rats. AAPS PharmSciTech.

[53] Köhler BM, Günther J, Kaudewitz D, Lorenz H-M (2019). Current therapeutic options in the treatment of rheumatoid arthritis. J Clin Med.

[54] Akhtar A (2015). The flaws and human harms of animal experimentation. Camb Q Health Ethics.

[55] Van Norman GA (2019). Limitations of animal studies for predicting toxicity in clinical trials: is it time to rethink our current approach?. JACC Basic Transl Sci.

[56] Reza Khorramizadeh M, Saadat F. Animal models for human disease. Anim Biotechnol. 2020:153–71. 10.1016/B978-0-12-811710-1.00008-2.

[57] Oliveira IM, Carvalho MR, Fernandes DC, Abreu CM, Maia FR, Pereira H, et al. Modulation of inflammation by anti-TNF α mAb-dendrimer nanoparticles loaded in tyramine-modified gellan gum hydrogels in a cartilage-on-a-chip model. J Mater Chem B. 2021. 10.1039/D1TB00802A. 10.1039/d1tb00802a 33998627

[58] Kuyinu EL, Narayanan G, Nair LS, Laurencin CT (2016). Animal models of osteoarthritis: classification, update, and measurement of outcomes. J Orthop Surg Res.

[59] Roberts I (2002). Does animal experimentation inform human healthcare? Observations from a systematic review of international animal experiments on fluid resuscitation. BMJ.

[60] Sandercock P, Roberts I (2002). Systematic reviews of animal experiments. Lancet.

[61] Pound P, Ebrahim S, Sandercock P, Bracken MB, Roberts I (2004). Where is the evidence that animal research benefits humans?. BMJ.

[62] Bahadoran Z, Mirmiran P, Kashfi K, Ghasemi A (2020). Importance of systematic reviews and meta-analyses of animal studies: challenges for animal-to-human translation. J Am Assoc Lab Anim Sci.

[63] Marx U, Akabane T, Andersson TB, Baker E, Beilmann M, Beken S, et al. Biology-inspired microphysiological systems to advance medicines for patient benefit and animal welfare in drug development. ALTEX. 2020. 10.14573/altex.2001241. 10.14573/altex.2001241 PMC7863570 32113184

[64] McNary SM, Athanasiou KA, Reddi AH (2012). Engineering lubrication in articular cartilage. Tissue Eng Part B Rev.

[65] Liu SQ, Tian Q, Hedrick JL, Po Hui JH, Ee PL, Yang YY (2010). Biomimetic hydrogels for chondrogenic differentiation of human mesenchymal stem cells to neocartilage. Biomaterials.

[66] Liu C, Constantinides PP, Li Y (2014). Research and development in drug innovation: reflections from the 2013 bioeconomy conference in China, lessons learned and future perspectives. Acta Pharmaceutica Sin B.

[67] Gupta N, Liu JR, Patel B, Solomon DE, Vaidya B, Gupta V (2016). Microfluidics-based 3D cell culture models: Utility in novel drug discovery and delivery research. Bioeng Transl Med.

[68] Fu L, Li P, Li H, Gao C, Yang Z, Zhao T (2021). The application of bioreactors for cartilage tissue engineering: advances, limitations, and future perspectives. Stem Cells Int.

[69] Meinert C, Schrobback K, Hutmacher DW, Klein TJ (2017). A novel bioreactor system for biaxial mechanical loading enhances the properties of tissue-engineered human cartilage. Sci Rep.

[70] Chen T, Buckley M, Cohen I, Bonassar L, Awad HA (2012). Insights into interstitial flow, shear stress, and mass transport effects on ECM heterogeneity in bioreactor-cultivated engineered cartilage hydrogels. Biomech Modeling Mechanobiol.

[71] Daly AC, Sathy BN, Kelly DJ. Engineering large cartilage tissues using dynamic bioreactor culture at defined oxygen conditions. J Tissue Eng. 2018;9:2041731417753718. 10.1177/2041731417753718 PMC5788092 29399319

[72] Vonk LA, van Dooremalen SFJ, Liv N, Klumperman J, Coffer PJ, Saris DBF (2018). Mesenchymal stromal/stem cell-derived extracellular vesicles promote human cartilage regeneration in vitro. Theranostics.

[73] Zhou G, Jiang H, Yin Z, Liu Y, Zhang Q, Zhang C (2018). In vitro regeneration of patient-specific ear-shaped cartilage and its first clinical application for auricular reconstruction. EBioMedicine.

[74] Chen Y, Ma M, Teng Y, Cao H, Yang Y, Wang Y (2020). Efficient manufacturing of tissue engineered cartilage in vitro by a multiplexed 3D cultured method. J Mater Chem B.

[75] Rosser J, Bachmann B, Jordan C, Ribitsch I, Haltmayer E, Gueltekin S (2019). Microfluidic nutrient gradient–based three-dimensional chondrocyte culture-on-a-chip as an in vitro equine arthritis model. Mater Today Bio.

[76] Goldman SM, Barabino GA (2017). Cultivation of agarose-based microfluidic hydrogel promotes the development of large, full-thickness, tissue-engineered articular cartilage constructs. J Tissue Eng Regenerative Med.

[77] Decante GBH, Costa J, Silva-Correia J, Collins M, Reis RL, Oliveira JM. Engineering bioinks for 3D bioprinting. Biofabrication. 2021. 10.1088/1758-5090/abec2c. 10.1088/1758-5090/abec2c 33662949

[78] Unagolla JM, Jayasuriya AC (2020). Hydrogel-based 3D bioprinting: a comprehensive review on cell-laden hydrogels, bioink formulations, and future perspectives. Appl Mater Today.

[79] Galarraga JH, Kwon MY, Burdick JA (2019). 3D bioprinting via an in situ crosslinking technique towards engineering cartilage tissue. Sci Rep.

[80] Xu J, Zheng S, Hu X, Li L, Li W, Parungao R (2020). Advances in the research of bioinks based on natural collagen, polysaccharide and their derivatives for skin 3D bioprinting. Polymers (Basel).

